# The challenge of localizing hyperkinetic seizures: timing matters!

**DOI:** 10.1055/s-0046-1827048

**Published:** 2026-08-13

**Authors:** İrem İlgezdi Kaya, Gulshan Aliyeva, Nur Gülce İşkan, Ayşe Deniz Elmalı, Nerses Bebek

**Affiliations:** 1 Istanbul University, Istanbul Faculty of Medicine, Department of Neurology, Division of Clinical Neurophysiology, Istanbul, Turkey.; 2 Istanbul University, Istanbul Faculty of Medicine, Department of Neurology, Istanbul, Turkey.; 3 Istanbul University, Center for Research in Epilepsy, Istanbul, Turkey.

**Keywords:** Hyperkinetic Movement, Epilepsy, Frontal Lobe, Epilepsy, Temporal Lobe, Electroencephalography

## Abstract

**Background:**

Hyperkinetic seizures (HSs), which are generally associated with the frontal lobe (although they may indeed originate from other regions too), are characterized by complex motor movements with high amplitude and short duration.

**Objective:**

To differentiate HSs originating from the frontal and extrafrontal lobes via electroencephalographic (EEG) features, notably timing.

**Methods:**

Clinical and video-EEG data of patients with HS were examined retrospectively. The localization of the ictal and interictal discharges, the onset and duration of the hyperkinetic movements, as well as the ictal-postictal electrophysiological and semiological features, were reviewed. The patients were divided into two groups according to their HS onset timing: if the onset of hyperkinetic semiology was in the first 10 seconds, they were classified as *early-onset HS* ; if later, as *late-onset HS* .

**Results:**

We included 29 patients (171 seizures); seizure onset was localized to the frontal region in 21 patients, and to the temporal and temporo-occipital regions in 6 patients, and it could not be determined in 2 patients. The HSs started in the early period in 21 patients, and in the late period in 8 patients; HSs originating from the frontal region started earlier than those originating from the temporal region (6.2 versus 25.7 seconds respectively; *p* = 0.002). Electrophysiological (35.4 versus 95.7 seconds respectively; *p* = 0.004) and clinical (34.3 versus 94.6 seconds respectively; *p* = 0.003) seizure durations were shorter in frontal-onset seizures compared with temporal-onset seizures.

**Conclusion:**

The timing of HS onset and the duration of the seizures are informative in terms of localization. Earlier onset of hyperkinetic semiology and shorter duration may point to a frontal origin.

## INTRODUCTION

Hyperkinetic seizures (HSs) consist of severe and inappropriate complex movements involving the proximal parts of the extremities and trunk. Initially described as *hypermotor seizures* by Lüders et al., 1 these are referred to as *hyperkinetic' seizures* in the 2006 report by the International League Against Epilepsy (ILAE) Classification Core Group. 2 Hyperkinetic semiology is observed in 15 to 17% of frontal-lobe epilepsies (FLEs) and is particularly associated with seizures originating from the mesio- and orbitofrontal regions. 3 This semiology can also be encountered in seizures originating from the temporal, insular, and parietal lobes. 4 5 6 7 The challenges posed by the complexity and heterogeneity of seizure semiology and clinical features, along with the typically-rapid spread of electrical discharge and artifacts, often complicate the interpretation of surface electroencephalography (EEG). 4 8 Therefore, understanding the origin of HSs is crucial to establish the relationship between semiology and cortical organization, in addition to EEG findings.

In the present study, the demographic, clinical, ictal, and postictal semiological, and electrophysiological characteristics of patients with HS were retrospectively reviewed to describe the differences between HSs originating from the frontal lobe and those from the extrafrontal lobes, particularly focusing on the usefulness of the timing of hyperkinetic semiology in localization efforts **.**

## METHODS

### Patients

In total, 982 video-EEG monitoring (VEM) reports from 2009 to 2022 were reviewed, and 29 patients whose VEM revealed HSs and had accessible recording data were included; thus, the demographic, clinical, and neuroimaging findings were obtained retrospectively from the patients' files.

### Demographic, clinical, and neuroimaging findings

As for the demographic data of the patients, age, sex, and age at the time of inclusion in the study were recorded. The clinical data was obtained from the medical records, and it included age at disease onset, duration of epilepsy, follow-up duration, seizure types (HS, other focal seizure, focal to bilateral tonic-clonic seizure, generalized seizure), seizure etiology, presence of drug-resistant epilepsy, history of seizure clustering (having ≥ 3 seizures within 24 hours), history of having > 1 seizure within 24 hours, currently- and previously-used antiseizure medication (ASM) and their numbers, whether epilepsy surgery was performed, history of head trauma, febrile convulsion, difficult birth, psychiatric disease, family history of epilepsy or febrile convulsion, presence of mental disability, and presence of abnormal neurological examination. To determine the area of seizure origin, EEG, semiology, brain magnetic resonance imaging (MRI), positron-emission tomography (PET), and neuropsychological test results (including the Luria sequential-drawings test, the Stroop test, the verbal-fluency test, the forward and backward digit-span tests, and the clock-drawing test) were used.

Seizure types, etiologies and epilepsy syndromes were classified according to the 2017 ILAE classification. 9 10 Additionally, previous EEG examinations in the records were reviewed, including interictal and ictal findings, background activity characteristics, ictal semiology, seizure localization (frontal and/or temporal), lateralization, neuropsychological test findings, brain MRI, and PET findings.

### EEG analysis and semiological features

Scalp EEG electrodes were placed according to the International 10-20 system during VEM. A total of 171 seizures from the patients' files were evaluated independently by 2 blinded researchers. The seizures were classified as *frontal* or *extrafrontal* based on the initial lobe of onset. The lobe of onset for the seizures and hyperkinetic movements was determined electrophysiologically or, if electrophysiological assessment was not possible (due to artifacts, rapid spreading etc.), semiologically. For frontal-lobe seizures, semiological features such as hypermotor activity, tonic posturing, rapid evolution, nocturnal predominance, and vocalizations were analyzed. For temporal-lobe seizures, auras, autonomic symptoms, behavioral arrest, automatisms, and postictal confusion were evaluated. Oroalimentary and manual automatisms were classified according to laterality. Additionally, asymmetrical tonic posturing and forced head deviation were systematically assessed. 1 11 Patients with non-localizable seizures (due to artifacts or generalized amplitude suppression) were noted. For patients experiencing HS and other types of seizures, those which were not HS were excluded from the evaluation. Additionally, seizures that could not be assessed due to technical issues (such as inadequate video resolution, no audio, or the patient being outside the camera's field of view during the seizure) were excluded. The presence of auras before the seizures was investigated. Postictal confusion was determined based on consciousness assessment conducted after seizure termination or by evaluating awareness status in video recordings. The time of day and whether the seizures occurred during sleep or wakefulness were reviewed. If the seizure occurred during sleep, the sleep stage at the time of the seizure was examined. The relationship between the onset time of ictal EEG activity and semiology was reviewed in terms of whether the EEG activity or the semiology started earlier. For semiological findings associated with the seizure, the period starting 10 seconds before the onset of ictal activity was systematically reviewed.

Hyperkinetic movements were classified as *early HS* if they occurred within the first 10 seconds of seizure onset, and as *late HS* if they started after 10 seconds following seizure onset. Ten minutes of data in the postictal period was systematically reviewed. 12 Postictal confusion was classified as *present* , *absent* , or *indeterminate* .

The ictal and postictal semiological and electrophysiological features evaluated included EEG findings such as lateralization, localization, and duration of the seizures, duration and timing of the hyperkinetic movements, and duration, presence, and timing of vocalization.

### Ethical approval and informed consent statements

The current study was approved by the Istanbul University Clinical Research Ethics Committee (Approval No. 2023/1456; approval date: 04 August 2023). Informed written consent, which enabled us to use their data in scientific studies, had been obtained from all patients prior to their admission for VEM, as this is our routine practice.

### Statistical analysis

Data was analyzed using the IBM SPSS Statistics for Windows (IBM CForp.) software, version 22.0. Descriptive statistics were used to calculate mean and standard deviation values. Group distributions and normality were assessed via the Shapiro-Wilk test. Independent group comparisons were performed with the Mann-Whitney-U tests. Categorical data were compared using the Chi-squared test.

## RESULTS

### Demographic and clinical features

A total of 29 patients were included in the study. Demographic and clinical features of the whole patient cohort are shown in Table 1 .

**Table 1:** Demographic and clinical features of the patient cohort

Features	Frontal (n = 21)	Extrafrontal (n = 6)	Total (n = 29)
Mean age (years)	35.8 ± 6.9	33.7 ± 10.1	35.2 ± 7.3
Sex: female/male (n)	9/12	2/4	11/18
Mean age at seizure onset (years)	12.5 ± 6.5	8.7 ± 6.3	11.2 ± 6.5
Risk factor for epilepsy in the medical history: n (%) Trauma; Febrile seizures	13 (61.9); 6 (28.6); 3 (14.3)	4 (66.7); 1(16.7); 3 (50)	19 (65.5); 7 (24.1); 6 (22.2)
Comorbidities: n (%) Developmental delay; Psychiatric disorders	10 (47.6); 4 (19); 6 (28.6)	2 (33.3); 2 (33.3); 0	14 (48.3); 8 (27.6); 6 (20.7)
Family history of epilepsy or febrile seizures: n (%)	9 (42.9)	2 (33.3)	12 (41.4)
**Brain magnetic resonance findings §**			
Normal: n (%)	12 (57.1)	1 (20)	14 (50)
Epilepsy-specific findings: n (%) Focal cortical dysplasia; Mesial temporal sclerosis; Focal cortical atrophy; Encephalomalacia	5 (23.8); 3; * 0; 1; # 1; †	4 (80); 2; ** 2; *** 0; 0	10 (35.7); 5; 2; 2; 1
Pathological, but not epilepsy-specific: n (%)	4 (19)	0	4 (13.8)
**Surgeries and outcomes: n**			
Resective surgery	1 ‡	0	Engel IIa
Vagal nerve stimulation implantations	3	0	Engel IIIa (n = 2) Engel IVc (n = 1)
**Clustering: n (%)**			
> 1 seizure within 24 hours	17 (81)	3 (50)	22 (75.9)
True clustering (≥ 3 seizures/day)	12 (57.1)	1 (16.7)	13 (44.8)

Notes: ^§^ Brain MRI was available for 28 patients, one patient underwent cranial CT because MRI was contraindicated;

* one patient with left medial frontal cortical dysplasia, one with right superior medial frontal gyrus cortical dysplasia, and one with left posterior frontal cortical dysplasia

** one patient with right temporal cortical dysplasia, and one with right frontal cortical dysplasia

*** one patient with left mesial temporal sclerosis, and one with right mesial temporal sclerosis

# one patient with bilateral frontal cortical atrophy

† one patient with left frontal encephalomalacia

‡ one patient with right frontal dysplasia

### EEG characteristics

#### *Seizure lateralization and localization*

When the EEG and semiological characteristics of the seizures were examined, the lateralization could not ve determined in 14 patients (48.3%). Of the remainder, seizures in 9 patients (31%) were lateralized to the right hemisphere, and, in 6 patients (20.7%), to the left hemisphere. Seizures were localized to the frontal lobe in 21 patients, in the temporal and temporoparieto-occipital regions in 6 patients, and they could not be determined in 2 patients. The demographic, semiological and electrophysiological characteristics of the patients based on seizure-onset localization are shown in Table 2 . As hypothesized, hyperkinetic semiology started earlier in the 21 patients with frontal lobe-onset seizures compared to the 6 patients with extrafrontal-onset seizures. Seizure duration, both semiologically and electrophysiologically, was also shorter in these patients. A patient consistently experiencing early-onset HS had an odds ratio (OR) of 30 for frontal onset seizures (95%CI: 3–394).

**Table 2:** Demographic, semiological and electrophysiological characteristics of the patients based on seizure-onset localization

Patients (N = 29)	Frontal (N = 21)	Extrafrontal (N = 6)	*p*
Age: median (IQR)	35.0 (12.0)	30.0 (9.8)	0.085
Sex (female:male)	8:13	2:4	0.613
Seizure duration electrophysiologically (seconds): median (IQR)	35.4 (28.3)	95.7 (37.4)	**0.004**
Seizure duration semiologically (seconds): median (IQR)	34.3 (25.1)	94.6 (48.8)	**0.003**
Duration of hyperkinetic movements (seconds): median (IQR)	25.8 (16.5)	17.2 (25.7)	0.550
Time between electrophysiologic onset andhyperkinetic movements (seconds): median (IQR)	6.2 (5.0)	25.7 (20.0)	**0.002**
Duration of postictal confusion (seconds): median (IQR)	0.0 (16.4)*	87.2 (120.4)**	0.079

Abbreviation: IQR, interquartile range.

Notes: *N = 14; **N = 4. Values of *p* in bold indicate statistical significance.

#### *Semiologic and electrophysiologic features of seizures*

The details regarding the 171 seizures examined, consisting of 152 frontal and 19 extrafrontal seizures, are shown in Table 3 . Earlier onset of hyperkinetic semiology and shorter duration of semiological and electrophysiological findings were consistently revealed for frontal lobe-onset seizures compared to extrafrontal ones. Similarly, each seizure of early-onset had an OR of 177.4 for frontal lobe onset (95%CI: 22.0–1430.7).

**Table 3:** Seizure Characteristics according to the lobe of origin

	All seizures (N = 171)	Frontal (N = 152)	Extrafrontal (N = 19)	*p*
Hyperkinetic semiology onset time (seconds): median (IQR)	4.0 (7)	4.0 (5)	24 (25)	**< 0.001**
Electrophysiological seizure duration (seconds): median (IQR)	32.0 (30)	27.5 (23)	100.0 (50)	**< 0.001**
Semiological seizure duration (seconds): median (IQR)	30.0 (32)	26.5 (24)	94.0 (48)	**< 0.001**
Hyperkinetic semiology duration (seconds): median (IQR)	20.0 (18)	20.0 (17)	37 (36)	0.175
Seizure time: n Day; Night	59; 112	51; 101	8; 11	0.455
Seizure during sleep: n (%)	76.0% (130/171)	78.3% (119/152)	57.8% (11/19)	0.082
Sleep stage of seizures: n Stage 1; Stage 2; Stage 3; Rapid eye movement; Awake	38; 81; 12; 0; 40	31; 78; 11; 0; 32	7; 3; 1; 0; 8	**0.020**
Postictal sleeping: n (%)	30.1% (46/153)	29.7% (41/138)	33.3% (5/15)	0.772
Postictal confusion (n) Yes; No; N/A	44; 62; 65	33; 59; 60	11; 3; 5	**0.003**
Duration of postictal confusion: median (IQR)	0.0 (297)	0.0 (25) *	110.0 (297) **	**0.043**
Postictal tachycardia: n (%)	50.5% (53/105)	47.8% (44/92)	69.2% (9/13)	0.165

Abbreviations: IQR, interquartile range; N/A, not available.

Notes: *N = 68; **N = 7. Values of *p* in bold indicate statistical significance.

#### *Seizure onset lobe and other electrophysiological/semiological characteristics*

Postictal confusion was significantly more frequent in extrafrontal seizures compared to frontal seizures ( *p* = 0.003). Furthermore, the duration of postictal confusion was notably longer in extrafrontal seizures than in frontal seizures ( *p* = 0.043), showing that extrafrontal seizures were associated with more prolonged and frequent postictal confusion compared to frontal seizures.

When analyzing the sleep stage of seizures, extrafrontal seizures occurred predominantly during wakefulness and less frequently during stage 1, whereas frontal seizures were more common in stage 2 ( *p* = 0.020). Although seizures during sleep were more common in both groups, frontal seizures exhibited a higher occurrence during sleep (78.3%) compared to extrafrontal seizures (57.8%), without reaching statistical significance ( *p* = 0.082).

When evaluating patients with more than 1 seizure within 24 hours, we found that they were older ( *p* = 0.037), had an earlier onset of hyperkinetic semiology ( *p* = 0.032), and experienced a shorter duration of postictal confusion ( *p* = 0.033). Electroencephalographic ( *p* = 0.001) and semiological ( *p* = 0.02) seizures were also shorter in patients with clustering.

### History and EEG findings

In patients with a history of head trauma, the hyperkinetic component ( *p* = < 0.001), and the durations of EEG ( *p* = 0.005) and semiological seizures ( *p* = 0.005) were shorter. Patients with a history of febrile seizures (FSs) presented a younger age at seizure onset ( *p* = 0.042) and longer durations of EEG ( *p* = 0.003) and semiological ( *p* = 0.003) seizures. The presence of FS history differed concerning the lobe in which the seizures initially occurred ( *p* = 0.043), with FS history being more common in cases of seizures originating from the temporal lobe and other regions compared to the frontal lobe. Consistently, the presence of FS history was inversely associated with clustering ( *p* = 0.008) and experiencing multiple seizures within 24 hours ( *p* = 0.038). History of difficult delivery was associated with younger age at seizure onset ( *p* = 0.036) and a higher number of previous ASMs ( *p* = 0.007).

## DISCUSSION

**Figure 1:**
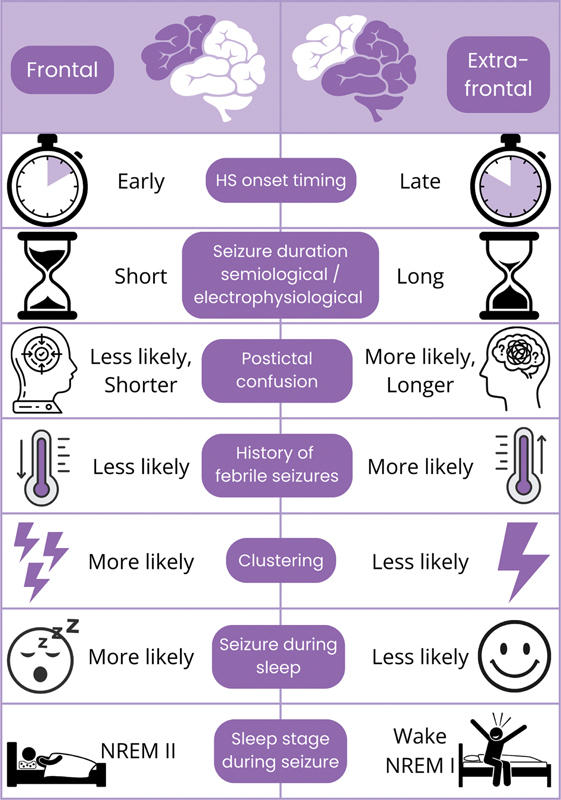
Multi-item, point-based infographic for the localization of hyperkinetic seizures.

Hyperkinetic seizures are known to predominantly originate from the frontal lobe, although they can also have extrafrontal origins. The current study revealed that the timing of the hyperkinetic component can help in differentiating between the frontal and extrafrontal seizure onset. An early onset of HS is a strong indicator of frontal onset, and a shorter seizure duration is an important supportive finding. We believe that, if a multi-item, point-based checklist can be developed, it would aid the epileptologist in difficult cases, and our suggestion is shown in Figure 1 .

In the current study, aligning with previous research, 3 13 14 HS predominantly originated from the frontal lobe, although the temporal and temporoparietal regions were also significant areas of origin. Apart from these regions, HSs have been reported 5 6 15 16 to originate from the insular, parietal, and parietotemporal regions, but we did not encounter any patients with insular or parietal origin in our cohort.

The differences noted in the timing and length of hyperkinetic semiology between frontal and extrafrontal seizures likely indicate distinct dynamics in seizure propagation and epileptogenic network organization. Semiology represents the clinical expression of seizure-related electrical activity arising from the interplay between seizure onset and early propagation, which are strongly shaped by underlying connectivity. 17 As clinical symptoms typically present several seconds after electrographic onset, the temporal characteristics of semiology may reflect early propagation patterns rather than just the anatomical site of origin. 17 The earlier onset and shorter duration of hyperkinetic behaviors observed in frontal-lobe seizures in the present study aligns with the rapid and multidirectional propagation supported by robust frontal-lobe connectivity, 18 19 in contrast with the more gradual evolution commonly observed in temporal-lobe seizures. 8 20 Surface video-EEG-based semiological analysis is still essential in the standard clinical practice, even though invasive methods such as stereo-EEG offer detailed network-level information. These findings imply that systematic evaluation of the timing of hyperkinetic semiology provides easily-accessible and clinically-relevant information that could support multimodal localization strategies.

The timing of hyperkinetic semiology is generally reported to be observed early in the course of an HS, regardless of localization in previous studies. Alqadi et al. 3 reported that hyperkinetic semiology usually appears within the first 10 seconds of clinical seizure onset, while Vaugier et al. 21 reported a delay of 4 to 11 seconds after ictal EEG onset. In the present study, the median time from electrographic seizure onset to hyperkinetic semiology was 4 (interquartile range [IQR]: 7) seconds. Nitta et al. 15 reported that hyperkinetic semiology occurs on average 5.3 seconds after seizure onset in FLE, and 11.6 seconds after seizure onset in temporal lobe epilepsy (TLE), but this difference was not statistically significant. Similarly, Alqadi et al. 3 found that the hyperkinetic component begins on average 2 seconds after ictal EEG onset in FLE, and 5 seconds after ictal EEG onset in seizures originating from extrafrontal lobes, without a statistically significant difference. However, we observed that the onset of hyperkinetic semiology in frontal seizures was significantly earlier compared to seizures originating from the temporal lobe. Another inference from the current study is that hyperkinetic semiology starting within the first 10 seconds may suggest a frontal lobe origin of the seizure, but it does not necessarily imply significant lateralization. Additionally, as in previous studies, 15 22 we found that shorter durations of electrographic and semiological seizures were noted in frontal-onset seizures.

The association of specific risk factors in medical history with an earlier onset of epilepsy has been reported in studies, suggesting that this finding is not specific to HS alone. Febrile seizure was more prevalent in cases of TLE compared to FLE, as expected. 23 24 Furthermore, both the electrographic and semiological seizure durations were longer in cases with a history of FS. This is likely due to the predisposition of FS in TLE cases and the longer seizure duration in TLE compared to FLE. 22 25

In addition to these findings, we noted that, in patients with a history of head trauma, the hyperkinetic component of seizures had shorter electrophysiological and semiological durations. Although the literature 26 27 reports FLE as the second most common type of epilepsy after TLE in traumatic brain injury, we found that a history of head trauma is more common in FLE compared to TLE in patients with HS. This may be due to underdiagnosis of FLE in traumatic brain injury cohorts, but it could also be a distinguishing finding in the HS patient group.

The current study has some limitations, such as the small number of patients in our cohort. Since extratemporal epilepsies are recognized less frequently and directed to tertiary centers for epilepsy surgery to an even lesser extent, the number of the patients in our cohort is scarce, although it spans a wide time range and a vast number of VEM studies. This challenge is further reinforced by the fact that HS is an extremely-rare semiological finding. Another limitation is the retrospective nature of the study. We believe these limitations may be overcome by collaboration efforts and building a multicenter database to collect data, ideally in a prospective manner. Such a study, incorporating tools such as ictal single-photon emission computed tomography (SPECT), invasive recordings, and data from surgery outcomes can more effectively address the challenges of localization and lateralization observed in VEM.

In conclusion, while HSs predominantly originate from the frontal lobe, they can also have extrafrontal origins. The present study revealed that the timing of the hyperkinetic component can help in differentiating between frontal and extrafrontal seizure onset. An early onset of HS is a strong indicator of frontal onset, and a shorter seizure duration is an important supportive finding.

## References

[1] Lüders H Acharya J Baumgartner C Semiological seizure classification Epilepsia 1998 39 09 1006 1013 10.1111/j.1528-1157.1998.tb01452.x 9738682

[2] Engel J Jr. Report of the ILAE classification core group Epilepsia 2006 47 09 1558 1568 10.1111/j.1528-1167.2006.00215.xPubMed 16981873

[3] Alqadi K Sankaraneni R Thome U Kotagal P Semiology of hypermotor (hyperkinetic) seizures Epilepsy Behav 2016 54 137 141 10.1016/j.yebeh.2015.11.017 26708064

[4] Staack A M Bilic S Wendling A S Hyperkinetic seizures in patients with temporal lobe epilepsy: clinical features and outcome after temporal lobe resection Epilepsia 2011 52 08 1439 1446 10.1111/j.1528-1167.2011.03100.x 21569022

[5] Ryvlin P Minotti L Demarquay G Nocturnal hypermotor seizures, suggesting frontal lobe epilepsy, can originate in the insula Epilepsia 2006 47 04 755 765 10.1111/j.1528-1167.2006.00510.x 16650142

[6] Nishibayashi H Ogura M Taguchi M Miki J Uematsu Y Itakura T Nondominant parietotemporal cortical dysplasia manifesting as hypermotor seizures Epilepsy Behav 2009 14 04 691 695 10.1016/j.yebeh.2009.02.010 19232546

[7] Wang L Mathews G C Whetsell W O Abou-Khalil B Hypermotor seizures in patients with temporal pole lesions Epilepsy Res 2008 82 01 93 98 10.1016/j.eplepsyres.2008.07.005 18760904

[8] McGonigal A Frontal lobe seizures: overview and update J Neurol 2022 269 06 3363 3371 10.1007/s00415-021-10949-0 35006387

[9] Fisher R S Cross J H D'Souza C Instruction manual for the ILAE 2017 operational classification of seizure types Epilepsia 2017 58 04 531 542 10.1111/epi.13671 28276064

[10] Scheffer I E Berkovic S Capovilla G ILAE classification of the epilepsies: Position paper of the ILAE Commission for Classification and Terminology Epilepsia 2017 58 04 512 521 10.1111/epi.13709 28276062 PMC5386840

[11] Riney K Bogacz A Somerville E International League Against Epilepsy classification and definition of epilepsy syndromes with onset at a variable age: position statement by the ILAE Task Force on Nosology and Definitions Epilepsia 2022 63 06 1443 1474 10.1111/epi.17240 35503725

[12] Whitehead K Gollwitzer S Millward H Wehner T Scott C Diehl B The additional lateralizing and localizing value of the postictal EEG in frontal lobe epilepsy Clin Neurophysiol 2016 127 03 1774 1780 10.1016/j.clinph.2015.11.050 26750581

[13] Menghi V Bisulli F Cardinale F Predictors of hyperkinetic seizures Epilepsy Behav 2022 129 108629 10.1016/j.yebeh.2022.108629 35272206

[14] Rheims S Ryvlin P Scherer C Analysis of clinical patterns and underlying epileptogenic zones of hypermotor seizures Epilepsia 2008 49 12 2030 2040 10.1111/j.1528-1167.2008.01675.x 18503559

[15] Nitta N Usui N Kondo A Semiology of hyperkinetic seizures of frontal versus temporal lobe origin Epileptic Disord 2019 21 02 154 165 10.1684/epd.2019.1047 31010798

[16] Jobst B C Gonzalez-Martinez J Isnard J The insula and its epilepsies Epilepsy Curr 2019 19 01 11 21 10.1177/1535759718822847 30838920 PMC6610377

[17] Bartolomei F Lagarde S Wendling F Defining epileptogenic networks: Contribution of SEEG and signal analysis Epilepsia 2017 58 07 1131 1147 10.1111/epi.13791 28543030

[18] Catani M Dell'acqua F Vergani F Short frontal lobe connections of the human brain Cortex 2012 48 02 273 291 10.1016/j.cortex.2011.12.001 22209688

[19] Bonini F McGonigal A Trébuchon A Frontal lobe seizures: from clinical semiology to localization Epilepsia 2014 55 02 264 277 10.1111/epi.12490 24372328

[20] Chowdhury F A Silva R Whatley B Walker M C Localisation in focal epilepsy: a practical guide Pract Neurol 2021 21 06 481 491 10.1136/practneurol-2019-002341 34404748

[21] Vaugier L Aubert S McGonigal A Neural networks underlying hyperkinetic seizures of “temporal lobe” origin Epilepsy Res 2009 86(2-3):200 208 10.1016/j.eplepsyres.2009.06.007 19619985

[22] Gibbs S A Proserpio P Francione S Seizure duration and latency of hypermotor manifestations distinguish frontal from extrafrontal onset in sleep-related hypermotor epilepsy Epilepsia 2018 59 09 e130 e134 10.1111/epi.14517 30009443

[23] Attumalil T V Sundaram A Varghese V O Vijayakumar K Kunju P A Risk factors of childhood epilepsy in Kerala Ann Indian Acad Neurol 2011 14 04 283 286 10.4103/0972-2327.91950 22346018 PMC3271468

[24] Vestergaard M Pedersen C B Sidenius P Olsen J Christensen J The long-term risk of epilepsy after febrile seizures in susceptible subgroups Am J Epidemiol 2007 165 08 911 918 10.1093/aje/kwk086 17267419

[25] Dash D Tripathi M The extratemporal lobe epilepsies in the epilepsy monitoring unit Ann Indian Acad Neurol 2014 17 01 S50 S55 10.4103/0972-2327.128655 24791090 PMC4001235

[26] Gupta P K Sayed N Ding K Subtypes of post-traumatic epilepsy: clinical, electrophysiological, and imaging features J Neurotrauma 2014 31 16 1439 1443 10.1089/neu.2013.3221 24693960 PMC4132580

[27] Lucke-Wold B P Nguyen L Turner R C Traumatic brain injury and epilepsy: Underlying mechanisms leading to seizure Seizure 2015 33 13 23 10.1016/j.seizure.2015.10.002 26519659 PMC12767287

